# Cyclical stretch induces structural changes in atrial myocytes

**DOI:** 10.1111/jcmm.12064

**Published:** 2013-04-26

**Authors:** Anne Margreet De Jong, Alexander H Maass, Silke U Oberdorf-Maass, Rudolf A De Boer, Wiek H Van Gilst, Isabelle C Van Gelder

**Affiliations:** a Department of Cardiology, University Medical Center Groningen, University of GroningenGroningen, The Netherlands; b The Interuniversity Cardiology Institute NetherlandsUtrecht, The Netherlands

**Keywords:** Atria, stretch, remodelling, cell culture

## Abstract

Atrial fibrillation (AF) often occurs in the presence of an underlying disease. These underlying diseases cause atrial remodelling, which make the atria more susceptible to AF. Stretch is an important mediator in the remodelling process. The aim of this study was to develop an atrial cell culture model mimicking remodelling due to atrial pressure overload. Neonatal rat atrial cardiomyocytes (NRAM) were cultured and subjected to cyclical stretch on elastic membranes. Stretching with 1 Hz and 15% elongation for 30 min. resulted in increased expression of immediate early genes and phosphorylation of Erk and p38. A 24-hr stretch period resulted in hypertrophy-related changes including increased cell diameter, reinduction of the foetal gene program and cell death. No evidence of apoptosis was observed. Expression of atrial natriuretic peptide, brain natriuretic peptide and growth differentiation factor-15 was increased, and calcineurin signalling was activated. Expression of several potassium channels was decreased, suggesting electrical remodelling. Atrial stretch-induced change in skeletal α-actin expression was inhibited by pravastatin, but not by eplerenone or losartan. Stretch of NRAM results in elevation of stress markers, changes related to hypertrophy and dedifferentiation, electrical remodelling and cell death. This model can contribute to investigating the mechanisms involved in the remodelling process caused by stretch and to the testing of pharmaceutical agents.

## Introduction

Atrial fibrillation is the most common cardiac arrhythmia 1 and it often occurs because of an underlying disease, such as hypertension or heart failure 2. Structural remodelling seen in patients with AF as well as in animal models includes atrial dilatation, cellular hypertrophy, dedifferentiation, fibrosis, apoptosis and myolysis 3–7. Electrophysiological changes also occur and may contribute to the progressive nature of AF 4, 8. The underlying disease is a major causal factor of atrial remodelling 5–7, 9–15. The common factor in these underlying disease states is haemodynamic and pressure overload of the ventricles and the atria which causes stretching. However, it is difficult to determine in animal models whether the critical mechanism is mechanical stress or neurohormonal activation. *In vitro*, stretching of ventricular cardiomyocytes has been associated with hypertrophy, increased calcineurin activity, apoptosis and release of reactive oxygen species 16–18. Stretching of atrial cells, however, is not well characterized. Atria from hypertensive animals show increased weight, fibrosis and inflammation 11, 13, 15 and long-term hypertension (4–5 years) results in enlarged atria, hypertrophy, fibrosis, dedifferentiation, cell death and myolysis, but without any sign of inflammation 5. In *in vitro* experiments, static stretch leads to electrical remodelling, hypertrophy, increased calcineurin activity and extracellular matrix remodelling 19–21. Static stretch, however, may not reflect the physiological situation.

In an effort to better simulate the clinical setting of atrial pressure overload, our objective was to develop an atrial cell culture model mimicking atrial remodelling due to atrial pressure overload.

## Materials and methods

### Ethics statement

All animal studies were conducted in accordance with the NIH Guide for the Care and Use of Laboratory Animals and approved by the Committee for Animal Experiments of the University of Groningen (Approval ID: DEC 5495 and DEC 6002).

### Isolation and culture of neonatal rat atrial cardiomyocytes

Neonatal rat atrial cardiomyocytes (NRAM) were isolated and cells were cultured, as described previously 22. Briefly, the hearts of 1- to 3-day-old Sprague Dawley rats were excised and transferred in a tube containing cold calcium- and bicarbonate-free Hanks with Hepes solution (CBFHH) (137 mmol/l NaCl, 5.36 mmol/l KCl, 0.81 mmol/l MgSO_4_, 5.55 mmol/l dextrose, 0.44 mmol/l KH_2_PO_4_, 0.34 mmol/l Na_2_HPO_4_ and 20 mmol/l HEPES, pH 7.4). Atria and ventricles were then separated and atrial cardiomyocytes were isolated by successive rounds of stirring and pipetting the tissue up and down in CBFHH supplemented with 1.5 mg/ml trypsin (BD Biosciences, Breda, the Netherlands) and 20 μg/ml DNase. To enrich the cardiomyocytes, the cell suspension was pre-plated on regular polystyrene dishes for 45 min. and the cardiomyocytes were then seeded on flexible collagen-coated silicone rubber membranes (Flexcell International Corporation, Hillsborough, NC, USA) coated with 0.1% gelatin at a density of 62500 cells/cm^2^.

Cardiomyocytes were cultured for 24 hrs at 37°C under 5% CO_2_ in MEM and supplemented with sodium bicarbonate for 5% CO_2_, 25 mmol/l Hepes (Invitrogen Corporation, Breda, the Netherlands), 1.5 mmol/l vitamin B12, 5% FCS, 0.1 mmol/l BrdU and penicillin-streptomycin (50 U/ml and 50 mg/ml, respectively; Invitrogen Corporation). The cells were then cultured in the same medium without serum and BrdU, but supplemented with transferrin (10 μg/ml), insulin (10 μg/ml) and bovine serum albumin (BSA; 1 mg/ml) for 18–48 hrs before the experiments were carried out.

Inhibitors were incubated with the cells for 30 min. prior to the stretch period. Concentrations used were: 1 μmol/l losartan, 1 μmol/l of the selective Ca^2+^/calmodulin-dependent protein kinase II (CaMKII) inhibitor KN93, 1 μmol/l cyclosporin A (Merck, Darmstadt, Germany), 10 μmol/l eplerenone and 10 μmol/l pravastatin.

### Application of mechanical stretching

Cardiomyocytes were stretched using the Flexercell-4000 system (Flexcell International Corporation), a modification of the system initially described by Banes *et al*. 23. Cyclical stretching of 1 Hz and 15% elongation was applied for different durations ranging from 30 min. to 24 hrs. Control cardiomyocytes were cultured on identical Flexerwell plates and maintained under similar conditions, but without mechanical stretch. Following the stretch periods, the cells were washed twice with PBS and used for protein analysis, immunofluorescence or RNA isolation.

### Cell diameter

Cells were fixed in 4% paraformaldehyde for 5 min. on ice, permeabilized with ice-cold PBS + 0.3% Triton X100 for 5 min. and then washed with PBS. A solution containing 3% BSA, 2% normal goat serum and 0.1% tween in PBS was used to block and reduce the background. Antibodies were diluted in 1% BSA in PBS and incubated with the cells for 1 hr at room temperature. The primary antibody was a mouse monoclonal anti-α-actinin (clone EA-53) and the secondary antibody was a goat antimouse FITC (Santa Cruz Biotechnology, Heidelberg, Germany). Counterstaining with fluorescent rhodamine-phalloidin (Invitrogen Corporation) was performed to differentiate non-cardiomyocytes. Coverslips were mounted using Vectashield mounting medium with DAPI (Vector Laboratories, Burlingame, CA, USA). Four to eight pictures were taken and 5–10 cells/picture were used per experiment. Image processing software (Image J 1.43u, NIH, Bethesda, MD, USA) was used to determine Feret's cell diameter. Cell diameter was expressed as a percentage of the diameter of control cells.

### Real-time quantitative PCR

Total RNA was isolated using TRIzol reagent (Invitrogen Corporation) or Nucleospin II kit (Macherey-Nagel, Düren, Germany) and converted to cDNA by QuantiTect Reverse Transcription (Qiagen, Venlo, the Netherlands). Gene expression was measured with Absolute QPCR SYBR Green ROX Mix (Abgene, Epsom, UK) in the presence of 7.5 ng cDNA and 200 nM forward and reverse primers. qRT-PCR was performed on the Biorad CFX384 (Bio-Rad, Veenendaal, the Netherlands). The initial denaturation and activation of the DNA polymerase (95°C for 3 min.) was followed by 35 cycles with denaturation for 15 sec. at 95°C and annealing and elongation for 30 sec. at 60°C followed by a melt curve. Gene expression levels were corrected for ribosomal protein, large, P0 (Rplp0) reference gene expression and values were expressed relative to the respective control group of each experiment. Primers used included: jun proto-oncogene (c-*jun*) forward gatcatccagtccagcaatg, c-*jun* reverse tattctggctatgcagttcag, FBJ osteosarcoma oncogene (c-*fos*) forward tccaagcggagacagatcaac, c-*fos* reverse tggcaatctcggtctgcaa, myelocytomatosis oncogene (c-*myc*) forward ctagtgctgcatgaagagac, c-*myc* reverse ttgctgtggcctcttgatgg, early growth response gene 1 (*egr-1*) forward gcacctgaccacagagtcctt, *egr-1* reverse ggtagtttggctgggataacttg, atrial natriuretic peptide (ANP) forward atgggctccttctccatcac, ANP reverse tctaccggcatcttctcctc, brain natriuretic peptide (BNP) forward acaatccacgatgcagaagct, BNP reverse gggccttggtcctttgaga, growth differentiation factor-15 (GDF15) forward tgacccagctgtccggatac, GDF15 reverse gtgcacgcggtaggcttc, α-myosin heavy chain (MHC) forward gacaactcctcccgctttgg, αMHC reverse aagatcacccgggacttctc, βMHC forward gtcaagctcctaagtaatctgtt, βMHC reverse gaaaggatgagcctttctttgc, skeletal α-actin (ACTA1) forward gggcaggtcatcaccatc, ACTA1 reverse tacaggtccttcctgatgtc, sodium channel, voltage-gated, type V, alpha subunit (Scn5a) forward ctgctcgtcatggtcattgg, Scn5a reverse tgaggttgtctgcgctgaag, α1c subunit of L-type Ca^2+^-channel (LTCC) forward ccggaagccagtgcatttt, LTCC reverse gttggtgaagatcgtgtcattgac, potassium voltage-gated channel, Shal-related subfamily, member 3 (Kcnd3) forward gccttgccagaatccgtgtg, Kcnd3 reverse gacgtggtcttgcccatgtg, potassium voltage-gated channel, KQT-like subfamily, member 1 (Kcnq1) forward gtttgaacagggtggaagac, Kcnq1 reverse tcatcactggctacgacttg, potassium inwardly rectifying channel, subfamily J, member 2 (Kcnj2) forward gcaatgccggagttcatatc, Kcnj2 reverse tgctaagtctctggcactac, inwardly rectifying channel, subfamily J, member 3 (Kcnj3) forward tgcgcaacagccacatggtc, Kcnj3 reverse cacgtggcaaatggtgagag, potassium intermediate/small conductance calcium-activated channel, subfamily N, member 1 (Kcnn1) forward ccggactgtgaagattgaac, Kcnn1 reverse ctcatatgcgatgctctgtg, solute carrier family 8 (sodium/calcium exchanger), member 1 (Ncx1) forward attgaagcgatcaccgtcag, Ncx1 reverse ggacgaaggcaaacagaacc, regulator of calcineurin 1 (Rcan1) forward agcgaaagtgagaccagggc, Rcan1 reverse ggcagggggagagatgagaa, Ca^2+^/calmodulin-dependent protein kinases IIδ (CaMKIIδ) forward aaacgcatcacagcctctga, CaMKIIδ reverse gaggcaacagtagaacgttgaca, angiotensin II receptor type 1a forward cacaaccctcccagaaagtg, angiotensin II receptor type 1a reverse tagagggtagggatcatgac, ras-related C3 botulinum substrate 1 (Rac1) forward aacctgcctgctcatcagtt, Rac1 reverse ttgtccagctgtgtcccata 24, inducible nitric oxide synthase (iNOS) forward ggaagaaatgcaggagatgg, iNOS reverse gcaggatgtcttgaacgtag, endothelial nitric oxide synthase (eNOS) forward tcctaacttgccttgcatcc, eNOS reverse ggcagccaaacaccaaagtc, Rplp0 forward gttgcctcagtgcctcactc and Rplp0 reverse gcagccgcaaatgcagatgg.

### Western blotting

Proteins from whole cell lysates were isolated using radioimmuno-precipitation assay buffer [0.5% sodium deoxycholate, 0.1% sodium dodecylsulfate (SDS) and 1% Igepal ca-630 in TBS] supplemented with a protease inhibitor cocktail (Roche Diagnostics Corp., Indianapolis, IN, USA), a phosphatase inhibitor cocktail, PMSF (1 mmol/l; Roche Diagnostics Corp.) and sodium vanadate (15 mmol/l). SDS sample buffer was added and samples were denaturized by heat at 99°C for 5 min. Proteins were separated by sodium dodecyl sulfate-polyacrylamide gel, transferred to PVDF membranes (Bio-Rad) and blocked with 5% milk in 0.1% tween in TBS (TBST). Blots were incubated overnight with antibodies against either phospho-Erk (E-4), troponin T-C (C-19) (both Santa Cruz Biotechnology), troponin I-C (MF4; Fitzgerald Industries International, Acton, MA, USA), phospho-Akt (Ser473) (D9E), phospho-p38 (Thr180/Tyr182; both Cell Signaling Technology, Danvers, MA, USA) or microtubule-associated protein light chain 3 (LC3; MBL, Naka-ku Nagoya, Japan) diluted in either 5% milk or BSA in TBST. For loading control, membranes were reprobed with α-tubulin or GAPDH antibodies (Fitzgerald Industries International). For loading phosphorylated proteins controls, the membranes were stripped (stripping buffer containing 2% SDS, 100 mmol/l β-mercaptoethanol and 62.5 mmol/l Tris buffer) for 30 min. at 55°C, washed with TBST and incubated with the corresponding antibody detecting the total protein amount, *i.e*. Erk 1/2 (MK1; Santa Cruz Biotechnology) and Akt (C67E7) and p38 (both Cell Signaling Technology). Signals were detected by ECL and quantified by densitometry (Syngene, Cambridge, UK). Protein levels were expressed relative to the respective control group of each experiment.

### ANP in cell culture medium

The level of ANP secreted into the medium was measured using a competitive enzyme immunoassay kit (Bachem, St. Helens, Merseyside, UK) according to the manufacturer's instructions. Samples were measured in duplicate and the levels of ANP were expressed as a percentage of ANP released by stationary cardiomyocytes.

### Cell death

To investigate whether apoptosis occurred, the terminal deoxynucleotidyl transferase (TdT)-mediated dUTP Nick End Labelling method (TUNEL) was used according to the manufacturer's instructions (Roche Diagnostics Corp.). Coverslips were mounted using Vectashield with DAPI (Vector Laboratories). To confirm that the TUNEL-labelled positive cells were cardiomyocytes, a counterstaining with α-actinin was performed, as described above.

To determine the amount of dead cells released into the cell culture medium, medium was taken together with PBS from two washes to include cells that were no longer fully attached. Cells were spun down, washed in PBS, collected in 200 μl PBS and counted (in duplicate) using a counting chamber (Fuchs-Rosenthal, Lo – Laboroptik Ltd., Lancing, UK). The amount of cells released into the medium was expressed relative to the amount of cells in the medium of stationary cells. Trypan blue staining was used to confirm that the majority of cells in the medium were dead cells.

In addition, lactate dehydrogenase (LDH) levels were determined in duplicate in cell culture medium using the Roche IFCC liquid assay on the modular analysis (Roche, Mannheim, Germany).

### Reagents and chemicals

Unless otherwise stated, all chemicals were purchased from Sigma-Aldrich Chemie B.V. (Zwijndrecht, the Netherlands).

### Statistics

Data are expressed as mean values ± S.E.M. Comparisons between groups were done using one-way anova with *post-hoc* Dunnett correction. For Figure 5b, a Bonferonni correction was used. Comparison of two groups was done using a two-tailed Student's *t*-test. All analyses were carried out using PASW (Version 18, SPSS Inc., Chicago, IL, USA). *P* values of <0.05 were considered statistically significant.

## Results

### Immediate early genes

**Fig. 1:**
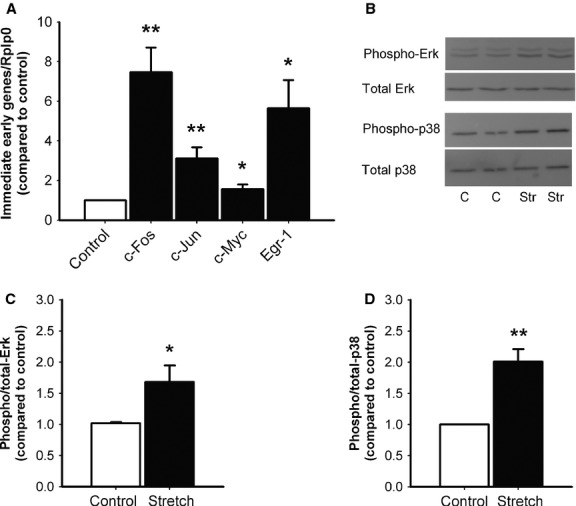
Stretching for 30 min. induces immediate early genes expression and phosphorylation of Erk and p38. (**A**) Expression of c-*fos*, c-*jun*, c-*myc* and *egr-1* upon stretching (*n* = 7). (**B**) Representative blots showing phosphorylated and total Erk and p38 levels after 30 min. of stretch. (**C**) Relative levels of phosphorylated Erk (*n* = 8). (**D**) Relative levels of phosphoryated p38 (*n* = 7). Data are expressed relative to control. *P < 0.05, **P < 0.01, compared with unstretched control. Egr-1: early growth response protein 1; Rplp0: ribosomal protein, large, P0; C: control; Str: stretch.

Stretch for 30 min. increased the mRNA expression of the immediate early genes c-*fos* by 647%, c-*jun* by 212%, c-*myc* by 56% and early growth response gene 1 (*egr-1*) by 465% (Fig. 1a). In addition, phosphorylation levels of Erk and p38 were increased 68% and 101% respectively (Fig. 1b–d).

### Hypertrophy and dedifferentiation

**Fig. 2:**
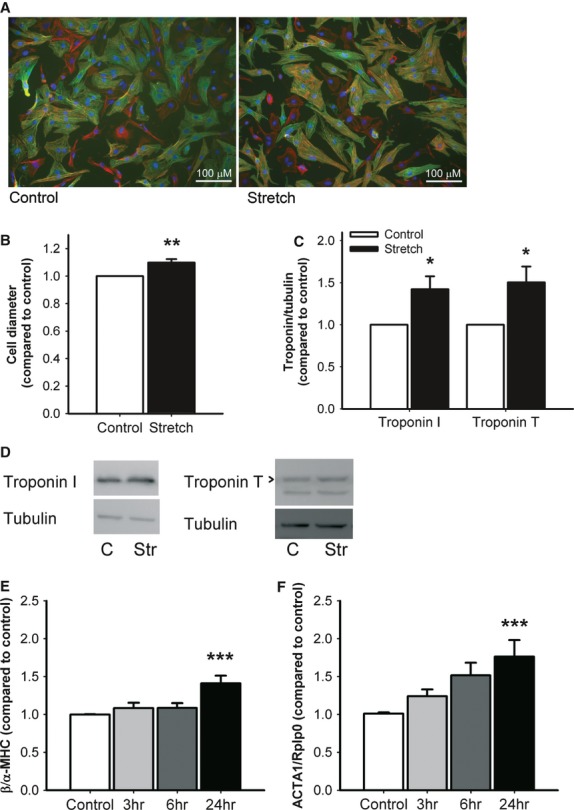
Stretching causes changes associated with hypertrophy and dedifferentiation. (**A**) Atrial myocytes were stained for α-actinin with specific antibodies (green) and actin was stained with Texas Red-phalloidin. Nuclei are stained blue with DAPI. (**B**) Relative cell diameter after 24 hrs of stretching (*n* = 11). (**C**) Relative protein levels of troponin I and troponin T (*n* = 18 and 19, respectively). (**D**) Representative blots showing levels of troponin I and troponin T after stretching for 24 hrs. (**E**) β/α-MHC ratio (*n* = 7–19). (**F**) Skeletal α-actin expression (*n* = 8–19). Data are expressed relative to control. *P < 0.05, **P < 0.01, ***P < 0.001 compared with unstretched control. C: control; Str: stretch; β/α-MHC: β/α-myosin heavy chain; ACTA1: skeletal α-actin; Rplp0; ribosomal protein, large, P0.

Stretch for 24 hrs increased the cell diameter by 10% (*P* < 0.05, Fig. 2a and b) along with troponin T and troponin I expression by 51% (*P* < 0.05) and 42% (*P* < 0.05), respectively, (Fig. 2c and d) suggesting hypertrophy-related increase in contractile protein expression. To investigate the dedifferentiation that often accompanies pathological hypertrophy, mRNA expression of skeletal α-actin, α-MHC and β-MHC were investigated. After 24 hrs of stretch, the β/α-MHC ratio and skeletal α-actin expression were increased by 41% and 76% respectively (Fig. 2e and f).

### Stress markers

**Fig. 3:**
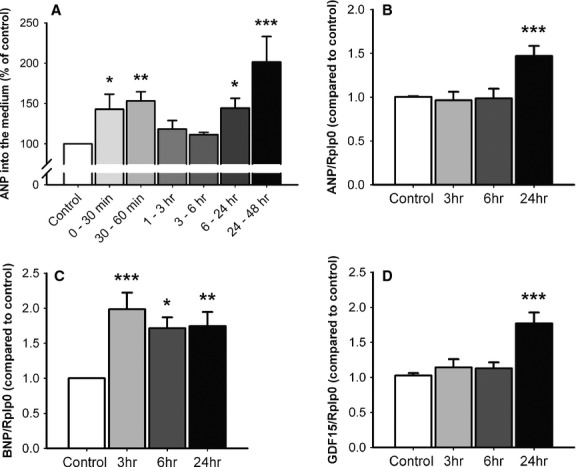
Stretching increases mRNA expression of stress specific markers and induces release of ANP. (**A**) ANP release into the medium (*n* = 3–9). (**B**) ANP expression (*n* = 8–21). (**C**) BNP expression (*n* = 8–20). (**D**) GDF15 expression (*n* = 8–19). Data are expressed relative to control. *P < 0.05, **P < 0.01, and ***P < 0.001 compared with unstretched control. ANP: atrial natriuretic peptide; BNP: brain natriuretic peptide; GDF15: growth differentiation factor-15; Rplp0: ribosomal protein, large, P0.

Stretch of NRAM caused a biphasic release of ANP (Fig. 3a), significantly different from control at 30 min., 1 hr, 24 and 48 hrs (increased by 43, 53, 44 and 102% respectively). Furthermore, 24 hrs of stretch increased mRNA levels of ANP by 47% (Fig. 3b). The biphasic response, with increased ANP mRNA expression, implies immediate release of ANP upon stretch, subsequent increased ANP production and a second phase of release of ANP. After 3 hrs of stretch, BNP mRNA expression levels were increased by 99% (Fig. 3c). Levels remained 70% increased after 6 and 24 hrs of stretch and the expression level of GDF15 was increased 77% after 24 hrs (Fig. 3d).

### Cell death

**Fig. 4:**
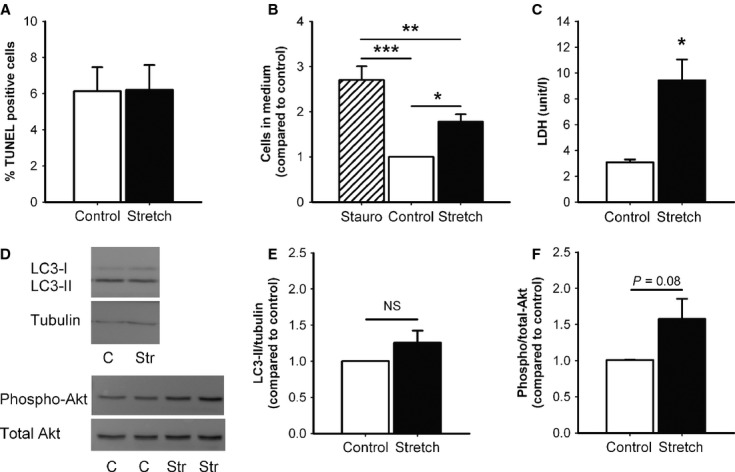
Stretching for 24 hrs induces cell death, but not *via* apoptosis or autophagy. (**A**) The amount of TUNEL-positive cells after 24 hrs of stretching (*n* = 11). (**B**) The relative amount of cells in the medium after stretching (*n* = 9–10). (**C**) The amount of LDH in the medium (*n* = 6). (**D**) Representative blots showing levels of LC3-II after 24 hrs of stretching and levels of phosphorylated and total Akt after 30 min. of stretching. (**E**) Relative protein levels of LC3-II (*n* = 10). (**F**) Relative levels of phosphorylated Akt (*n* = 8). Data are expressed relative to control. *P < 0.05, **P < 0.01, and ***P < 0.001 compared with unstretched control. PC: positive control, staurosporine (500 nM 24 hrs); LC3: microtubule-associated protein light chain 3.

Three different forms of cell death exist: apoptosis, cell death with autophagy and necrosis. TUNEL staining showed 6.1% TUNEL-positive cells in control cells compared with 6.2% following stretch (Fig. 4a). Western blotting for LC3-II, a marker of autophagosomes (a double-membrane structure which contains elements of its own cytoplasm mediating autophagy), revealed no difference between control cells and stretched cells (Fig. 4d and e). Although no sign of cell death was shown by TUNEL staining or LC3-II Western blotting, a 78% increase (Fig. 4b) in the amount of cells in the medium was observed after 24 hrs of stretch. As a positive control for cell death, staurosporine was used; staurosporine increased the amount of cells in the medium 170% (Fig. 4b). Trypan blue staining was used to confirm that the cells released in the medium were indeed dead cells. The majority of the cells (95%) stained blue, showing that these cells did not have an intact cell membrane. In addition, LDH levels were threefold increased in medium from stretched cells (Fig. 4c) Also, a trend towards increased phosphorylation of Akt, a protein involved in cellular survival pathways, was observed (Fig. 4d and f).

### Electrical remodelling

**Fig. 5:**
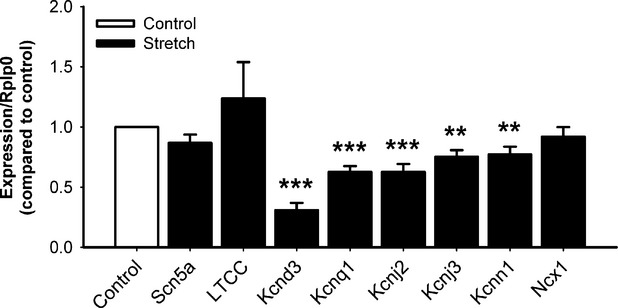
Changes in gene expression related to electrical remodelling. Effect of 24 hrs stretch on gene expression of Scn5a, LTCC, Kcnd3, Kcnq1, Kcnj2, Kcnj3, Kcnn1 and NCX1 (*n* = 9–11). Data are expressed relative to control. *P < 0.05, **P < 0.01 and ***P < 0.001 compared with unstretched control. Scn5a, sodium channel, voltage-gated, type V, alpha subunit; LTCC, α1c subunit of L-type Ca^2+^-channel; Kcnd3, potassium voltage-gated channel, Shal-related subfamily, member 3; Kcnq1, potassium voltage-gated channel, KQT-like subfamily, member 1; Kcnj2, potassium inwardly rectifying channel, subfamily J, member 2; Kcnj3, inwardly rectifying channel, subfamily J, member 3; Kcnn1, potassium intermediate/small conductance calcium-activated channel, subfamily N, member 1; Ncx1, solute carrier family 8 (sodium/calcium exchanger).

Figure 5 shows the effects of 24 hrs of stretch on genes related to electrical remodelling. Stretch for 24 hrs had no effect on expression of Scn5a which contributes to the sodium current (I_Na_), expression of LTCC which contributes to the L-type calcium current (I_CaL_) or expression of the sodium/calcium exchanger (Ncx1; Fig. 5). Stretch reduced expression levels of Kcnd3 to 30% of control levels. Kcnd3 encodes Kv4.3 and contributes to the cardiac transient outward potassium current (I_to_). Also expression of Kcnq1, Kcnj2 and Kcnj3 were reduced to 63, 63 and 75% of control levels, respectively. Kcnq1 encodes K_v_7.1 and mediates slow delayed rectifying K^+^ (I_Ks_), Kcnj2 codes for Kir2.1 and mediates the inward rectifier current (I_K1_) and Kcnj3 codes for Kir3.1 and mediates acetylcholine-activated inward rectifier K^+^ current *(*I_KACh_). Interestingly, also the expression of small conductance Ca^2+^-activated K^+^ channel 1 (SK1) was reduced.

### Mechanisms

**Fig. 6:**
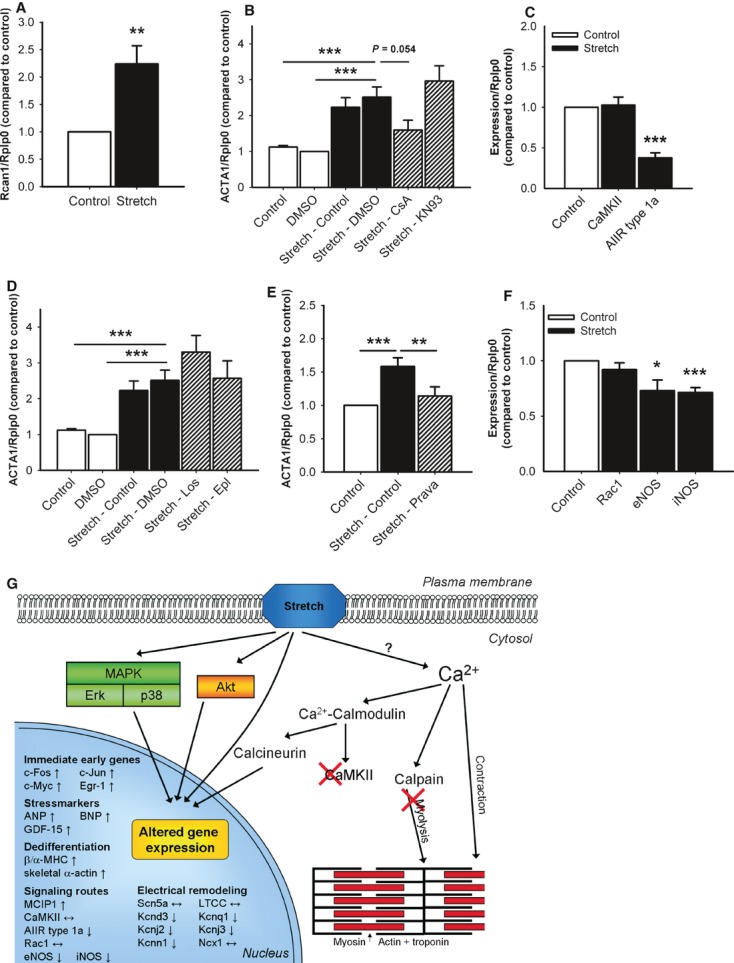
Mechanisms involved in the stretching response. Effect of 24 hrs of stretching on (**A**) Rcan1 expression (*n* = 19). (**B**) The effect of 1 μmol/l KN93 and 1 μmol/l cyclosporin A on the stretch-induced increase in skeletal α-actin expression (*n* = 7–18). (**C**) Expression levels of CaMKIIδ (*n* = 11), and expression of angiotensin II receptor type 1a (*n* = 8). (**D**) The effect of 1 μmol/l losartan and 10 μmol/l eplerenone on the stretch-induced increase in expression of skeletal α-actin (*n* = 5–18). (**E**) The effect of 10 μmol/l pravastatin on the expression of skeletal α-actin (*n* = 7). (**F**) Expression levels of Rac1, eNOS and iNOS (*n* = 9). (**G**) Overview of stretch-activated mechanisms. Data in A, C and G are expressed relative to control, in B, D, E and F data are expressed relative to unstretched control with 0.1% DMSO. *P < 0.05, **P < 0.01 and ***P < 0.001 compared with unstretched control and in Figure C, D, E and F compared with stretch with DMSO. Rcan1: regulator of calcineurin 1; Rplp0: ribosomal protein, large, P0; ACTA1: skeletal α-actin; GDF15: growth differentiation factor-15; DMSO: dimethyl sulfoxide; CsA: cyclosporin A; Epl: eplerenone; Los: losartan; Prava: pravastatin; CaMKII: Ca^2+^/calmodulin-dependent protein kinases IIδ; AIIR type 1a: angiotensin II receptor type 1a; Rac1: ras-related C3 botulinum substrate 1; eNOS: endothelial nitric oxide synthase; ANP: atrial natriuretic peptide; BNP: brain natriuretic peptide; β/α-MHC: β/α-myosin heavy chain; Egr-1: early growth response protein 1.

Stretch for 24 hrs increased the expression of Rcan1 by 124% (Fig. 6a) suggesting the involvement of calcineurin signalling. Furthermore, treatment with 1 μM cyclosporine A was associated with a trend towards attenuation of the stretch-induced increase in the expression of skeletal α-actin (Fig. 6b). This suggests activation of calcineurin signalling upon stretching and involvement in the stretch-induced response.

We could not demonstrate the involvement of CaMKII in the stretch-activated response as no increase in expression of CaMKIIδ, the major CaMKII isoform in the heart, was observed after 24 hrs of stretch (Fig. 6c) and inhibition of CaMKII with 1 μM KN93 did not alter the expression level of skeletal α-actin (Fig. 6b).

### Interventions

Treatment with losartan had no effect on the gene expression of skeletal α-actin (Fig. 6d) which suggests that this response is not mediated *via* ligand activation of the angiotensin II type 1 receptor. However, the expression of the angiotensin II receptor type 1a was decreased (Fig. 6c). Incubation with eplerenone had no effect on stretch-induced gene expression, but pravastatin reduced stretch-induced expression of skeletal α-actin (Fig. 6e). No effect of stretch on expression of Rac1 was observed (Fig. 6f), but the expression of eNOS and iNOS was reduced (Fig. 6f).

## Discussion

This study shows that cyclic stretch of NRAM results in (1) activation of immediate early genes, distinct signalling pathways, changes related to hypertrophy and dedifferentiation, elevation of stress markers, electrical remodelling and cell death; (2) activation of calcineurin signalling, but not of CaMKII signalling; (3) inhibition of skeletal α-actin expression with pravastatin and (4) the demonstration that BNP in atrial cells is a stronger stress marker than ANP in neonatal atrial cardiomyocytes *in vitro*.

Activation of immediate early genes is an early response to a wide variety of growth factors, cytokines as well as injury. Previous studies in isolated rat atria showed that stretch increased the expression of c-*fos*, c-*myc* and *egr-1*, but not of c-*jun* 25, 26 which is in agreement with our study. In ventricular cells, activation of immediate early genes has also been demonstrated in response to stretch 27, 28. Kerkela *et al*. showed a rapid increase in phosphorylated p38 and Erk due to stretch which is in agreement with our data showing that 30 min. of stretch increased phosphorylation levels of p38 and Erk 26. Similar findings have also been reported in stretched ventricular cardiomyocytes 17, 29.

In atrial cardiomyocytes subjected to stretch, hypertrophy was suggested *via* an increase in protein to DNA ratio and ANP mRNA levels 19, 20. We also observed an increase in cell length, troponin expression and the expression levels of ANP, which is often used as a marker of hypertrophy. In animal models of hypertension, enlarged atria have also been observed 5, 11, 13, 15, 30–32, as well as atrial dedifferentiation, in models of hypertension, mitral valve disease and atrial fibrillation 5, 32, 33 which is in agreement with the increased levels of gene expression markers of dedifferentiation we observed in our study. An important aspect of dedifferentiation is myolysis, which has been described in cells subjected to tachypacing and in patients with AF 34, but has not been described in association with stretch. Reduced expression of troponins was found in AF patients, which correlated with the amount of myolysis 35. We, however, did not observe any reduction in troponin protein levels which suggests that stretch for 24 hrs is a model in which remodelling takes place without the loss of contractile elements.

GDF15 is a potential new biomarker and a 25-fold increased expression has been observed in stretched ventricular myocytes 36. In our experimental model, we only observed a 1.8-fold increase in expression. This discrepancy may be due, at least in part, to the different stretch protocols used (cyclic *versus* static).

Cell death has not been described in stretched atrial cardiomyocytes. In hypertensive sheep, atrial apoptosis has been demonstrated 5, but in a dog model of mitral regurgitation, no sign of necrosis was found 31. We observed an increase in cell death which probably did not occur *via* apoptosis because there was no increase in TUNEL-positive cells. In addition, no protective effect of the autophagosome was found (unchanged protein levels of LC3-II).

Electrical remodelling has been described extensively related to AF 37, 38. In cells, Yang *et al*. showed that rapid electrical stimulation of HL-1 cells induced electrical remodelling, including shortening of action potential duration 39. In patients with hypertension, electrical remodelling has also been described 9. More recently, also SK1 and SK2 were recognized to play a role in electrical remodelling in AF 40. In atrial cardiomyocytes stretch also been related to electrical remodeling. Saygili *et al*. found a reduced I_to_ and a reduced expression of Kv4.2, in addition, an increased I_K1_ and an increased expression of Kir2.1 and Kir2.3 was observed [19]. These findings are partially in agreement with ours; we observed a reduced expression of Kv4.3 and also a reduced expression of Kir2.1.

Many changes in calcium handling and Ca^2+^-dependent mechanisms are related to atrial remodelling 41. Static atrial stretch has been shown to increase calcineurin activity and Rcan1 expression 20. Furthermore, cyclosporine A, a calcineurin inhibitor, reduced stretch-induced changes in extracellular matrix remodelling in atrial cardiomyocytes 21. Our observations are in agreement with these previous studies. We observed a stretch-induced increased Rcan1 mRNA expression, and cyclosporine A tended to attenuate the increase in skeletal α-actin expression. In AF, down-regulation of the expression of the L-type Ca^2+^-channel has been observed 42. Similarly, reduced atrial L-type Ca^2+^-current has been demonstrated in patients with mitral valve disease or reduced ejection fraction 43. However, we did not observe any reduction in L-type Ca^2+^-channel expression after 24 hrs of stretch.

In an atrial cardiomyocytes model of static stretch, losartan attenuated the stretch-induced hypertrophy and electrical and extracellular matrix remodelling 19, 21. In our experiments, losartan had no effect on stretch-induced increase in skeletal α-actin expression. We did, however, observe a down-regulation of the angiotensin II type 1 receptor which might be protective. An important difference between these studies and ours is the type of stretch used. In our experimental model, we used a similar elongation, but a cyclical stretch pattern which, as opposed to static stretch, mimics a more physiological condition. Furthermore, we could not show protective effects of eplerenone, but pravastatin prevented the stretch-induced increase in skeletal α-actin expression. This suggests a role for reactive oxygen species which has not been described in atrial cells. However, in ventricular myocytes, stretch has been shown to induce the release of reactive oxygen species and these reactive oxygen species mediated the effect of stretch 17, 44. Rac1 is a small GTPase that regulates the activity of NAPDH oxidase, producing superoxide. Increased expression of Rac1 has been implicated in AF 45. However, we did not observe changes in the expression of Rac1, but we did observe reduced eNOS and iNOS expression.

As expected, ANP expression, a key factor in cardiac stress, was increased upon stretch. We observed a comparable small increase in ANP mRNA levels as previously described in atrial cardiomyocytes 19, 20. Increased expression of BNP has also been found following atrial stretch 26. In human atrial strips, BNP is also induced by ischaemia; this is independent of mechanical stress 46. In our experiments, changes in BNP mRNA levels were more pronounced than changes in ANP expression levels. Our findings confirm that atrial cardiomyocytes are a source of BNP and show, *in vitro*, that stretch induced BNP expression. Interestingly, our results suggest that during stretch, BNP is a better stress marker than ANP in neonatal atrial cardiomyocytes *in vitro*.

In summary, we have demonstrated that cyclical stretch of atrial cardiomyocytes results in activation of immediate early genes and distinct signalling pathways including phosphorylation of Erk and p38, changes related to hypertrophy and dedifferentiation, elevation of stress markers such as ANP, BNP and GDF15, changes related to electrical remodelling and cell death (Fig. 6g). Previous studies demonstrated that static stretch results in hypertrophy 19, 20, increased MMP2 and MMP9 activity 21 and changes in potassium currents and gene expression suggesting electrical remodelling 19, 20. We observed the involvement of calcineurin, but not of CaMKII signalling. Incubation with losartan or eplerenone was without effect, but pravastatin reduced skeletal α-actin expression upon stretch. The role of calcineurin 20, 21 and angiotensin II-dependent signalling pathways has been suggested in previous studies 19, 21. Importantly, our results were obtained using cyclical stretch, a context reflecting more adequately the physiological condition where the pressure in the atrium increases during each heart cycle.

In ventricular cardiomyocytes, more data are available on the effects of *in vitro* stretch 16–18, 27, 28, 36, 47. In ventricular cardiomyocytes another method of stretch, using glass microspheres to induce mechanical load, did not induce hypertrophy, but did induce arrhythmias and caused the release of angiogenic factors 48. Results obtained using ventricular cardiomyocytes cannot be directly extrapolated because atrial and ventricular cardiomyocytes are anatomically and functionally different and express genes to a different extent 49, 50. In a microarray performed by Barth *et al.,* differences in gene expression were shown between human atria and ventricles 51. In the atria, genes associated with fibrosis, apoptosis and neurohormonal activation were more highly expressed whereas, in the ventricles, genes associated with the contractile function were more abundant 51.

## Limitations

We used cyclical stretch as a model of atrial overload caused by disease states that create a substrate for AF. It is not known whether the mechanisms described in our experimental model could be extrapolated to humans. We tested a limited number of mechanisms and signalling pathways that are often activated in ventricular disease as well as mechanisms that have been described in atrial fibrillation. It is likely that other signalling pathways are also activated, but this warrants further investigation. *In vitro*, stretch of atrial and ventricular cardiomyocytes can be dissected. *In vivo,* however, atrial fibrillation results in a high ventricular rate which has been shown to be an important causal factor of atrial remodelling 6, 10.

## Conclusions

The present stretch model resulted in changes mimicking the situation caused by underlying disease. Our model used cyclical stretch of the atrial cardiomyocytes and provides useful data on different aspects of remodelling. This model can be used to investigate mechanisms involved in the remodelling process as well as to assess the effect of new pharmaceutical agents on the remodelling process.
